# A PheWAS study of a large observational epidemiological cohort of African Americans from the REGARDS study

**DOI:** 10.1186/s12920-018-0462-7

**Published:** 2019-01-31

**Authors:** Xueyan Zhao, Xin Geng, Vinodh Srinivasasainagendra, Ninad Chaudhary, Suzanne Judd, Virginia Wadley, Orlando M. Gutiérrez, Henry Wang, Ethan M. Lange, Leslie A. Lange, Daniel Woo, Frederick W. Unverzagt, Monika Safford, Mary Cushman, Nita Limdi, Rakale Quarells, Donna K. Arnett, Marguerite R. Irvin, Degui Zhi

**Affiliations:** 1 Department of Biostatistics, University of Alabama at Birmingham, Birmingham, AL 35233 USA; 2 BGI-Shenzhen, Shenzhen, 518083 China; 3 School of Biomedical Informatics, The University of Texas Health Science Center at Houston, Houston, TX 77030 USA; 4 Department of Epidemiology, University of Alabama at Birmingham, Birmingham, AL 35233 USA; 5 Department of Medicine, University of Alabama at Birmingham, Birmingham, AL 35233 USA; 6 Department of Emergency Medicine, University of Alabama at Birmingham, Birmingham, AL 35233 USA; 7 Division of Biomedical Informatics and Personalized Medicine, Department of Medicine, University of Colorado Anschutz Medical Campus, Aurora, CO 80045 USA; 8 Department of Neurology and Rehabilitation Medicine, University of Cincinnati College of Medicine, Cincinnati, OH 45267 USA; 9 Department of Psychiatry, Indiana University School of Medicine, Indianapolis, IN 46202 USA; 10 Division of General Internal Medicine, Weill Cornell Medical College, Cornell University, New York, NY 10065 USA; 11 Department of Medicine and Pathology, Larner College of Medicine at the University of Vermont, Burlington, VT 05405 USA; 12 Department of Neurology, University of Alabama at Birmingham, Birmingham, AL 35294 USA; 13 Cardiovascular Research Institute, Department of Community Health and Preventive Medicine, Morehouse School of Medicine, Atlanta, GA 30310 USA; 14 College of Public Health, University of Kentucky, Lexington, KY 40506 USA; 15 School of Public Health, The University of Texas Health Science Center at Houston, Houston, TX 77030 USA

**Keywords:** PheWAS, African Americans, Genetics, Cardiovascular disease

## Abstract

**Background:**

Cardiovascular disease, diabetes, and kidney disease are among the leading causes of death and disability worldwide. However, knowledge of genetic determinants of those diseases in African Americans remains limited.

**Results:**

In our study, associations between 4956 GWAS catalog reported SNPs and 67 traits were examined among 7726 African Americans from the REasons for Geographic and Racial Differences in Stroke (REGARDS) study, which is focused on identifying factors that increase stroke risk. The prevalent and incident phenotypes studied included inflammation, kidney traits, cardiovascular traits and cognition. Our results validated 29 known associations, of which eight associations were reported for the first time in African Americans.

**Conclusion:**

Our cross-racial validation of GWAS findings provide additional evidence for the important roles of these loci in the disease process and may help identify genes especially important for future functional validation.

**Electronic supplementary material:**

The online version of this article (10.1186/s12920-018-0462-7) contains supplementary material, which is available to authorized users.

## Background

Genome Wide Association Studies (GWASs) have provided a powerful approach for identifying association between genetic variants and a single phenotype. An alternative and complementary approach to query genotype-phenotype associations is the Phenome-Wide Association Study (PheWAS) [1]. With PheWAS, associations between a specific genetic variant and a wide range of phenotypes can be explored. They are well suited to facilitate the identification of new associations between SNPs and phenotypes as well as SNPs with pleiotropy [2–4]. The PheWAS approach was mainly pioneered by investigators at Vanderbilt University [1] and flourished in various hospital-based cohorts by scanning phenomic data in electronic medical records for genetic associations [1, 4–6] as well as by meta-analyzing data collected in observational cohort studies like the Population Architecture using Genomics and Epidemiology (PAGE) study [2].

As of January 2017, GWASs have identified ~ 44,000 SNPs important for various human phenotypes as summarized in the GWAS catalog [7], which makes it possible to reveal pleiotropic effects and genetic mechanisms shared by different traits. Conducting PheWASs using SNPs which were reported to be associated with one or more traits is an efficient method for replication of previous results and identification of pleiotropic effects.

In this study, we used the REasons for Geographic And Racial Differences in Stroke (REGARDS) Study to examine 4956 GWAS catalog SNPs (Additional file 1) that are included on the Infinium HumanExome-12v1-2_A (exome chip) array from Illumina with a rich collection of phenotypes. The REGARDS study is a population-based, longitudinal study including 30,000 participants (~ 40% African Americans), sampled from the continental US [8]. Among 12,000 African American participants, 7726 were genotyped with the exome chip. Since most PheWAS studies have considered individuals of European ancestry and cross-sectional phenotypes, REGARDS is an excellent resource for both cross-racial validation and identifying pleiotropic effects.

## Results

We tested for association between 4956 GWAS catalog SNPs and 67 phenotypes. Genomic inflation factors (λ) generated from including all SNPs for a given phenotype showed good fitting of all models with λ range from 0.95 to 1.12. Table 1 summarizes 29 significant associations passing the significance threshold with *P* value less than 1.5E-7. S2 compares results extracted from the GWAS catalog on significant PheWAS SNPs to the REGARDS results. The significant associations are in several major phenotype groups: C reactive protein, lipid profile, diabetes, cystatin C, heart event risk, heart rate, and height. We classified the significant SNPs in two ways: 1. the SNP was associated to a phenotype matching previous publications 2. the SNP was associated to a phenotype related to the previously reported phenotype (Additional file 2).

**Table 1:** Summary of identified significant associations in REGARDS study

SNP ID	Phenotype	Minor allele (effect allele)	Major Allele	Beta or OR	P-value	MAF	First reported in AAs
Matched phenotype							
rs10096633	Triglycerides	T	C	− 0.020	4.88E-10	0.4226	
rs1173727	Height	T	C	0.297	9.89E-08	0.2032	yes
rs12110693	Heart rate	G	A	−1.302	4.28E-11	0.4984	
rs12740374	LDL Cholesterol	T	G	−4.314	1.64E-10	0.2615	
rs173539	HDL Cholesterol	T	C	2.337	1.21E-19	0.3647	yes
rs1800775	HDL Cholesterol	C	A	−2.843	1.53E-29	0.4272	yes
rs247616	HDL Cholesterol	T	C	4.309	4.88E-52	0.2528	yes
rs2794520	C reactive protein	T	C	−0.125	3.92E-34	0.2146	
rs326	Triglycerides	A	G	0.019	8.20E-09	0.4436	
rs3764261	HDL Cholesterol	A	C	3.050	1.84E-30	0.3165	
rs6511720	LDL Cholesterol	T	G	−5.624	1.19E-10	0.1337	
rs6511720	Total Cholesterol	T	G	−6.143	3.14E-10	0.1337	
rs7412	LDL Cholesterol	T	C	−15.870	2.17E-65	0.1114	
rs7499892	HDL Cholesterol	T	C	−2.351	1.38E-19	0.3677	yes
rs7553007	C reactive protein	A	G	−0.122	6.61E-34	0.2258	
rs876537	C reactive protein	T	C	−0.124	7.99E-33	0.2083	
rs9398652	Heart rate	C	A	−1.339	1.19E-11	0.4956	
Related phenotype							
rs12740374	Dyslipidemia	T	G	0.783	1.08E-10	0.2615	
rs12740374	Total Cholesterol	T	G	−4.152	3.24E-08	0.2615	
rs247616	Fram_CHD	T	C	−0.041	3.78E-09	0.2528	yes
rs629301	Dyslipidemia	G	T	0.827	4.32E-08	0.3633	
rs646776	Dyslipidemia	C	T	0.827	4.41E-08	0.3622	yes
rs6511720	Dyslipidemia	T	G	0.737	4.45E-10	0.1337	
rs7412	Fram_CHD	T	C	−0.066	3.03E-12	0.1114	
rs7412	Ideal7	T	C	0.210	3.35E-14	0.1114	
rs7412	Dyslipidemia	T	C	0.525	6.16E-33	0.1114	
rs7412	Total Cholesterol	T	C	−13.330	2.90E-37	0.1114	
rs7903146	Diabetes	T	C	1.306	2.30E-12	0.2919	
rs911119	Cystatin C	C	T	−0.012	6.17E-08	0.356	yes

Beta coefficients were showed for continuous variables and odd ratios (OR) were showed for binary variables. MAF: minor allele frequency. Matched phenotype means the same phenotype and SNP associations have been showed in previous published studies; if similar or related associations have been published before, they are defined as “related phenotype”. If this is the first time that an association was shown in Africa American population, “Yes” was given in the column” First reported in AAs “

### Validation of known genetic associations of phenotypes

Among the 29 significant genotype and phenotype associations, 17 have been previously reported for the same phenotype (Table 1 and Additional file 2). The effect directions of the 17 associations were the same as those in the previous reports. For eight of these phenotype –genotype associations, our study represents the first validation in an African American population (see section below). These replications validated the reliability of our PheWAS analysis approaches. We confirmed that C reactive protein level was related to rs2794520 (*P* = 3.9E-34), rs7553007 (*P* = 6.6E-34) and rs876537 (*P* = 8.0E-33), which are located near the *CRP* gene (Table 1). Five SNPs located near the *CETP* gene were associated with HDL cholesterol including rs173539 (*P* = 1.2E-19), rs1800775 (*P* = 1.5E-29), rs247616 (*P* = 4.9E-19), rs3764261 (*P* = 1.8E-30), and rs7499892 (*P* = 1.4E-19). Two SNPs were significantly associated with heart rate: rs12110693 near *LOC644502* gene (*P* = 4.3E-11) and rs9398652 near *GJA1* gene (P = 1.2E-11). We also reproduced the association between rs1173727 near the *NPR3* gene and height with *P* = 9.9E-8. Three SNPs were significantly associated with LDL cholesterol including rs12740374 in the *SORT1*/ *PSRC1*/ *CELSR2* cluster (*P* = 1.6E-10), rs6511720 in *LDLR* (P = 1.2E-10), and rs7412 in *APOE* (*P* = 2.2E-65). Rs10096633 in the *LPL* gene (P = 4.9E-10) and rs326 in the *C8orf35/SLC18A1*/*LPL* cluster (*P* = 8.2E-9) were associated with total cholesterol. Apart from 17 reported associations, the other 12 SNPs were associated with phenotypes that are closely related to previously published associations indexed in the GWAS catalog (Table 1 and Additional file 2).

### Cross-racial validation

Eight of our findings were reported in other races previously but not in African Americans. Observed associations of rs173539, rs1800775, rs247616, and rs7499892 with HDL had not been previously reported in African Americans. The other new cross-ethnic validations from our study included rs1173727 with height, rs911119 with cystatin C, rs247616 with the Framingham risk score, and rs646776 with dyslipidemia (Table 1 and Additional file 2). Interestingly, we saw even more significant results for the association between rs247616 and HDL with *P* = 4.88E-52 and beta value = 4.3 (mg/dL) in REGARDS, compared to *P* = 9.7E-24 and beta value = 3.0 (mg/dL) in the GWAS catalog report [9] (Additional file 2).

### SNPs associated with multiple traits

**Fig. 1:**
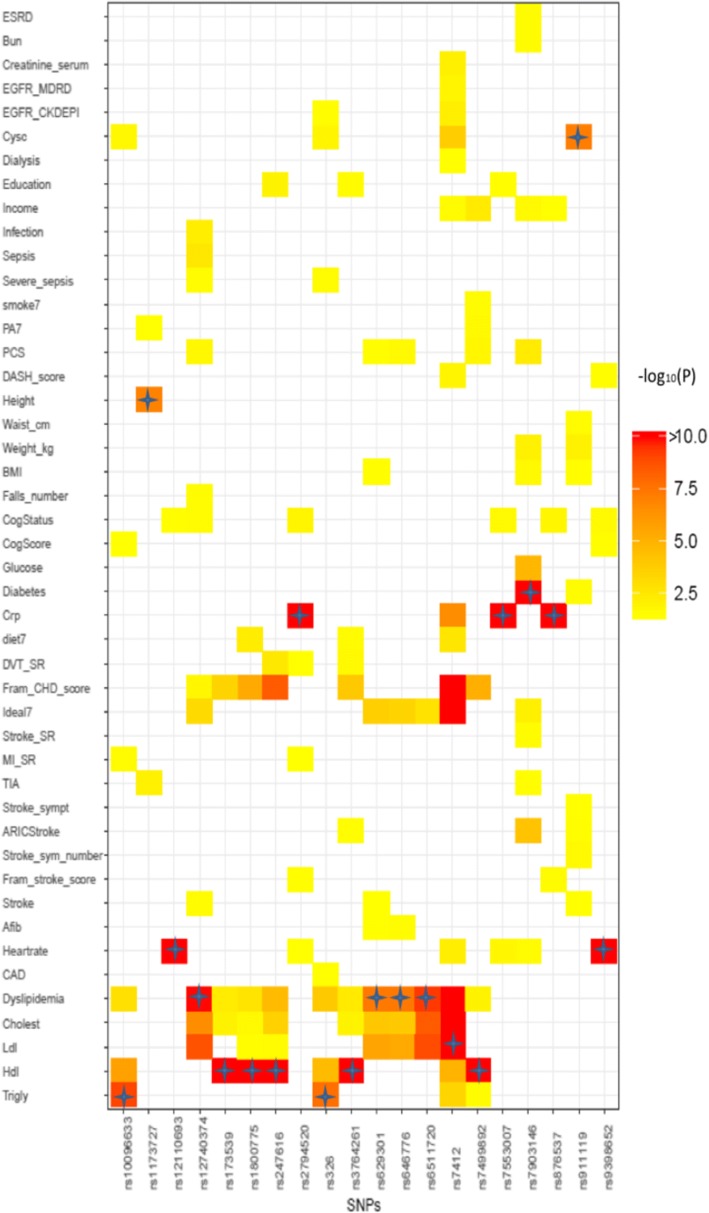
Heatmap shows the -log_10_P for association between SNPs with different traits. Shown in colors are the association *P* values of SNPs which are associated with first trait with *P* < 1.00E-7 and second trait with *P* < 0.05. The stars indicate the primary trait associated with the SNPs

The 29 significant genotype and phenotype associations involved 20 SNPs, and 11 of these were associated with multiple traits (*P*-value < 1.0E-7 for the first trait and *P* < 3.7E-5 for the second trait) (Additional file 3). We also listed the genome-wide significant SNPs for one trait which were suggestively associated with another trait with nominal *P* < 0.05 in Additional file 3. Figure 1 listed those 11 SNPs and another 8 SNPs which were significantly associated with the first trait (P-value < 1.0E-7) and nominally associated with another trait (P < 0.05). Generally, the pleotropic effects were caused by one SNP associated with multiple correlated phenotypes. In the conditional analysis, the associations were not significant between the second top traits and the corresponding SNPs after including the top traits as the covariate. For example, rs7412 was associated with LDL (*P* = 7.64E-62) and Cystatin C (*P* = 1.80E-04) due to a significant association between these two phenotypes (*P* = 6.48E-06).

## Discussion

Our PheWAS presented association of 4956 SNPs with 67 phenotypes using a subset of African Americans from the REGARDS study. Our study validated 29 previous GWAS associations, of which eight associations were reported for the first time in African Americans (AAs). Among many of our findings, 11 SNPs were associated with multiple traits.

We identified 29 significant genotype and phenotype associations. 17 of these have been reported previously. The phenotypes of the other 12 associations were related with those previously reported but not exactly the same. For instance, rs911119 located in the *CST3*/*CST4*/*CST9* gene cluster was reported previously associated with chronic kidney disease in a European population [10]. Our current study found that in African Americans allele C of rs911119 was negatively associated with the level of cystatin C, which is a biomarker for kidney function (*P* = 6.2E-8). Rs7903146 in *TCF7L2* gene was reported associated with type 2 diabetes in several different populations [11], which agrees with our current results (*P* = 2.3E-12). Rs247616 in the *CETP* gene was significantly associated with the Framingham CHD Hard Event Risk Score (Fram_CHD: Risk of Coronary Death or MI over 10 Years) with *P* = 3.8E-9. While this SNP has not been previously associated with the Framingham risk score, it has been associated with its components as well as related phenotypes including blood metabolite levels, cardiovascular disease risk factors, and lipoprotein-associated phospholipase A2 mass and activity only in Europeans [9, 12, 13]. Rs7412 in the APOE gene was associated with Fram_CHD (*P* = 3.0E-12), total cholesterol (*P* = 2.9E-37), lipidemia (P = 6.2E-33) and Ideal7 (the American Heart Association’s “Life’s Simple Seven” score, i.e., total number of ideal risk behaviors or metrics for each of the seven) (*P* = 3.3E-14). Our findings were consistent with previous studies, which showed that rs7412 was associated with several lipid related phenotypes including LDL cholesterol, lipid metabolism phenotypes, lipid traits, and response to statin therapy [14–17]. Here, we also found that rs629301 (in *CELSR2*, *PSRC1* and *SORT1*), rs646776 (in *CELSR2*, *PSRC1* and *SORT1*) and rs6511720 (in *LDLR*) are associated with dyslipidemia. This is in alignment with previously findings: associations of rs629301 with total cholesterol and LDL cholesterol [18]; associations of rs646776 with total cholesterol, LDL cholesterol, lipid metabolism phenotypes, coronary artery disease, myocardial infarction (early onset), and response to statin therapy in Europeans [19, 20]; associations of rs6511720 with total cholesterol, LDL cholesterol, lipid metabolism phenotypes, lipoprotein-associated phospholipase A2 activity and mass, and cardiovascular disease risk factors [18]. Rs12740374 in *CELSR2/PSRC1/SORT1* cluster was associated with two lipid traits: total cholesterol and dyslipidemia in our study, which is closely related with previously reported associations with LDL cholesterol and lipoprotein-associated phospholipase A2 activity and mass [21, 22].

We validated eight associations in AAs for the first time. Due to the difference of genetic variants between African Americans and the other races [23], it is interesting to check whether the associated variants reported in other races are associated with the same traits in AAs or not. When SNPs replicate across diverse populations, the gene’s importance in the disease process is emphasized, and consistency of findings may indicate genes that are especially important for future functional validation. Importantly, the effects of eight variants in AAs were of the same directions as in the other reported races.

## Conclusions

In this study, we leveraged the rich phenotype collection and the exome chip data in 7726 REGARDS AA participants, and examined the associations between 4956 GWAS catalog SNPs and 67 phenotypes. We validated 29 previous GWAS associations, of which eight associations were reported for the first time in AAs.

## Methods

### Study population and design

The REGARDS Study is a prospective, longitudinal population-based cohort study [8] of European American and African American adults aged 45 and older. Detailed description of the objectives and design of this study has been published [8]. The baseline telephone interview and separate in-home visit were conducted between 2003 to 2007 [24]. Baseline data collection resulted in a broad range of demographic, diet, and clinical information as well as banked biospecimens which were used to extract DNA and assess multiple clinical measurements [8]. Participants continue to be contacted every 6 months by telephone to identify stroke events and other incident outcomes [8]. The REGARDS study protocol was approved by the institutional review boards of each participating institution, and written informed consents were obtained from all participants. This current study examined phenotypes available in REGARDS participants to explore their association with exome-chip SNP genotypes. A total of 7726 self-reported African Americans with exome chip data were included in our study. The average age of participants was 64.6 years old (standard deviation = 9.0), and 4770 (61.7%) were female.

### SNP selection and genotyping

Genotyping was conducted using the Infinium HumanExome-12v1-2_A from Illumina (San Diego, CA, USA). The Illumina exome chip provides genotype data on > 240,000 putative functional variants selected based on over 12,000 individual exome and whole-genome sequences derived from individuals of European, African, Chinese, and Hispanic ancestry (http://genome.sph.umich.edu/wiki/Exome_Chip_Design). Raw genotyping data were called by GenomeStudio (version 2.0). The variant quality control included removing SNPs with call rate < 95%, monoallelic SNPs, multiallelic SNPs, and SNPs that had mapping errors. After further removing first and second degree relatives, samples with technical issues, and samples with mismatched sex, 7726 samples were available for analysis. In total, 4956 autosomal SNPs with minor allele frequency > 0.05 aligned to the GRCh37 reference sequence were matched to GWAS published SNPs catalog V1.0.1, which were reported to be associated with at least one trait with *P* < 1.0E-5 (Additional file 1) [7, 25].

### Phenotypes

Lists of phenotypes included in this study are shown in Table 2 and Table 3. The phenotypes included both baseline and incident events among the 7726 African Americans. Baseline information included medical history, personal history, demographic data, socioeconomic status, cognitive screening, laboratory assays, urine, height, weight, waist circumference, blood pressure, pulse, electrocardiography, and medications in the past 2 weeks [8]*.* Follow-up events included stroke, coronary heart disease (CHD), myocardial infarction, infection, sepsis, end-stage renal disease, and death. All the phenotypes were binary or continuous variables (See Tables 2-3). Totally, 26 binary and 41 continuous phenotypes were included for current study [26–68]. The binary variables follow a binomial distribution and their frequencies for each category were calculated. Most of the continuous variables followed normal distribution. For variables with large skewness or kurtosis, a logarithm or square root transformation was performed. Obvious outliers with values at more than 10 standard deviations away from the mean were redefined as missing.

**Table 2:** List of binary phenotypes

Short name	Category	Full description	Number of “yes”	Number of samples	Frequency of “yes” (%)
Prevalent Phenotypes					
CogStatus [26, 27]	Aging	Cognitive Status: Normal: defined as cogscore> 4, Impaired: defined as cogscore <=4	744	6195	12.01
Falls [28]	Aging	Self-reported fall in the past year	1166	7704	15.13
Afib [29, 30]	CVD related	Atrial Fibrillation (self-report or ECG evidence)	573	7526	7.61
CAD [31]	CVD related	History of Heart Disease (self-reported MI, CABG, bypass, angioplasty, or stenting OR evidence of MI via ECG	1186	7582	15.64
DVT [32]	CVD related	Self-reported deep vein thrombosis	371	7699	4.82
Hypertension [33, 34]	CVD related	Hypertensive if SBP > =140 or DBP > =90 or self-reported current medication use to control blood pressure	5622	7714	72.88
Dyslipidemia [35]	CVD related	Dyslipidemia: if TC > =240 or LDL > =160 or HDL < =40 or on medication	4171	7604	54.85
MI_SR [31]	CVD related	History of Myocardial Infarction (MI) (self-reported MI OR evidence of MI via ECG	891	7588	11.74
PAD_amputation [36]	CVD related	History of leg amputation	40	7725	0.52
PAD_surgery [36]	CVD related	Self-reported procedure to fix the arteries in legs	162	7709	2.1
Stroke_SR [37, 38]	CVD related	Participant reported stroke at baseline	597	7701	7.75
Stroke_Sympt [39, 40]	CVD related	Presence of stroke symptoms at baseline	1632	7134	22.88
TIA [29, 37]	CVD related	Participant reported Transient ischemic attack at baseline	257	7102	3.62
Diabetes [41]	Diabetes related	Diabetic if fasting glucose> = 126/non-fasting glucose> = 200 or pills or insulin	2335	7639	30.57
Cancer [42]	Other	Have you ever been diagnosed with cancer	526	4895	10.75
Orthopnea [29]	Other	Require more than one pillow to sleep at night	1076	7702	13.97
Dialysis [43]	Renal	Self-reported dialysis	45	7670	0.59
KidneyFailure [43]	Renal	Self-reported kidney failure	164	7670	2.14
Incident Phenotypes					
CHD [44]	CVD related	Incidence of coronary heart disease until 2012	436	7726	5.64
MI [44]	CVD related	Incidence of myocardial infarction until 2012	284	7726	3.68
Stroke [45]	CVD related	Incidence of Stroke until 20,150,401	287	7726	3.71
Death [46]	Other	Incidence of Death until 20,150,401	1494	7726	19.34
Infection [47, 48]	Other	Incidence of infection	548	7726	7.09
Sepsis [47, 48]	Other	Incidence of sepsis	307	7726	3.97
Severe_sepsis [47, 48]	Other	Incidence of severe sepsis	243	7726	3.15
ESRD [49]	Renal	Incidence of end stage renal disease until 2012	238	7726	3.08

**Table 3:** The list of continuous phenotypes of this study

Short name	Category	Full description	Data transformation	Number of samples	Mean	Standard deviation
CogScore [26, 27]	Aging	Computed cognitive score		6195	5.45	0.85
Falls_number [28]	Aging	Number of times fallen in the past year	log10(x + 1)	1182	0.42	0.2
MCS [50]	Aging	The mental component of the short-form 12 health survey: Mental		7352	53.46	9.02
BMI [51]	Body size	Body Mass Index - kg/m2		7657	30.84	6.73
Height [51]	Body size	Height		7702	66.4	3.88
Waist_cm [51]	Body size	Waist circumference (cm)		7673	98.43	15.42
Weight_kg [51]	Body size	Weight (kg)		7694	87.99	20.54
ARICStroke	CVD related	ARIC Stroke Risk Score: 10 Year Probability of Ischemic Scroke (%)	log10	6791	0.83	0.47
Cholest [52]	CVD related	Total Cholesterol (mg/dL)		7676	193.1	40.9
Crp [53]	CVD related	C reactive protein (mg/L)	log10	7597	0.46	0.52
DBP [54, 55]	CVD related	Diastolic blood pressure - average of two measures (mmHg)		7703	78.58	10.11
Fram_CHD_score [56]	CVD related	Framingham CHD Hard Event Risk Score: Risk of Coronary Death or MI over 10 Years (among those free of CHD at baseline)	log10	6381	0.86	0.4
Fram_stroke_score [57]	CVD related	Framingham Stroke Risk Score: 10 Year Probability of Stroke (%) (among those who self-reported never having a stroke at baseline)	log10	6694	0.88	0.39
Hdl [52]	CVD related	HDL Cholesterol (mg/dL)		7622	53.46	15.9
Heartrate [58]	CVD related	Heart rate (beats per minute)		7627	68.48	11.95
Ideal7 [59]	CVD related	American Heart Association Life simple seven, total number of ideal for each of the seven		7726	2.12	1.08
Ldl [52]	CVD related	LDL Cholesterol (mg/dL)		7566	116.81	36.42
SBP [54, 55]	CVD related	Systolic blood pressure - average of two measures (mmHg)		7703	131.41	17.29
SLFS [60]	CVD related	Family risk score for stroke		4293	−0.48	0.33
Stroke_Sym_Number [39, 40]	CVD related	Number of stroke symptoms		7134	0.39	0.87
Trigly [52]	CVD related	Triglycerides (mg/dL)	log10	7673	2.01	0.2
Glucose [41]	Diatetes related	Glucose (mg/dL from labs formerly from fromVermont)	sqrt	7676	10.38	1.78
Insulin [41]	Diatetes related	Endogenous Insulin uU/mL	log10	5619	1.09	0.35
CESD [61]	Other	Center for Epidemiologic Studies Depression Scale		7670	1.39	2.21
DASH_Score [62]	Other	DASH style diet score		4592	23.11	4.25
Diet7 [59]	Other	Life simple seven, diet score		4592	1.17	0.37
Education [63]	Other	1 = ‘Less than high school’; 2 = ‘High school graduate’; 3 = ‘Some college’; 4 = ‘College graduate and above’; missing = − 9.		7718	2.57	1.08
Income [63]	Other	Income		6763	5.7	2.13
MedDietScore [64]	Other	Mediterranean diet score		4483	4.43	1.64
PA7 [59]	Other	Life simple seven, physical activity		7618	1.89	0.79
PCS [50]	Other	PCS-12: SF-12 Physical	square root	7325	4.55	1.1
Smoke7	Other	Life simple seven, smoking		7726	2.63	0.76
TV [65]	Other	watching TV time. 0 = ‘None’; 1 = ‘1–6 h/wk’; 2 = ‘1 h/day’; 3 = ‘2 h/day’; 4 = ‘3 h/day’; 5 = ‘4+ hrs/day’; missing = − 9.		5408	3.81	1.39
ACR [66]	Rental	Urinary Albumin/Creatinine ratio (mg/g)	log10	7421	1.09	0.62
Albumin_urine [66]	Rental	Urinary albumin (mg/L)	log10	7423	1.2	0.63
BUN [66]	Rental	Blood-urea-nitrogen (mg/dL)	log10	5472	1.18	0.16
Creatinine_serum [67]	Rental	IDMS Calibrated Creatinine (mg/dL)	log10(x + 1)	7674	0.29	0.09
Creatinine_urine [66]	Rental	Urinary creatinine (mg/dL)		7437	152.1	84.59
Cysc [67]	Rental	Cystatin C (mg/L)	log10	7597	0	0.14
EGFR_CKDEPI [68]	Rental	estimated GFR from the CKD-Epi equation		7674	87.52	23.67
EGFR_MDRD [68]	Rental	Glomerular Filtration Rate (mL/min/1.73 square meters) using IDMS calibrated creatinine and MDRD equation		7674	89.36	27.15

### Statistical methods

Single SNP linear or logistic regressions were performed by PLINK for continuous or binary phenotypes respectively using an additive genetic model. The top 10 principal components determined by EIGENSTRAT [69], age, and gender were used as covariates for all phenotypes. Additional covariates were used for cholesterol, high-density lipoprotein (HDL), low-density lipoprotein (LDL), triglyceride, glucose, and insulin. Those covariates included whether the participants were fasted under examination, whether they had self-reported diabetes and took insulin/glucose lowering pills, and whether they had self-reported dyslipidemia and took lipid lowering medication.

The threshold of significance level for PheWASs is not straightforward and multiple approaches have been used in other PheWAS studies [2–4]. The PAGE study used five population-based studies representing major racial/ethnic groups, and their threshold is “ P<0.01 observed in two or more PAGE studies for the same SNP, phenotype class, and race/ethnicity, and consistent direction of effect” [2]. The Environmental Architecture for Genes Linked to Environment (EAGLE) study used similar threshold with an additional condition for allele frequency > 0.01 and sample size > 200 [4]. The Norfolk Island study performed a principal component analysis of phenotypes and used principal components as the final phenotypes. A *P* value of 1.84E-7 was considered the threshold for a significant association between a component and SNP [3]. In our study, the criteria for a significant association between a single SNP and a single phenotype with Bonferroni correction was defined as P value = $\frac{0.05}{4956\ast 67}$=1.5E-7. In our study, significant genotype and phenotype associations involved 20 SNPs. Therefore, the significance threshold for a second trait of the pleiotropic effect is *P* = 0.05/(67*20) = 3.7E-5.

## Additional files

Additional file 1: List of 4956 SNPs included in the association tests. (XLS 3240 kb)

Additional file 2: Title: Matching of Regard significant associations with Published GWAS catalog (XLSX 85 kb)

Additional file 3: SNPs associated with multiple traits (XLSX 45 kb)

## Abbreviations

GWAS: Genome-Wide Association Study
HDL: High-Density lipoprotein
LDL: Low-Density Lipoprotein
PAGE: Population Architecture using Genomics and Epidemiology
PheWAS: Phenome-Wide Association Study
REGARDS: REasons for Geographic and Racial Differences in Stroke
SNP: single nucleotide polymorphism
